# Fear of Recurrence and Medication Adherence in Pediatric Leukaemia Survivors in Low-Resource Settings

**Published:** 2026-03-26

**Authors:** SUDIP SAHA, AATEKA FATIMA MOHAMMED, DEEPTHI NAGULA, POOJA CHAKRABARTI, MAKURIRA VUNYOROHWASHE, SIRAJUDHEEN ANWAR, KAUSHALENDRA MANI TRIPATHI, SHAUMILE HASAN KHAN, KHWAJA WALEED MAQBOOL, KANWAL SHAZIA

**Affiliations:** 1 Department of Surgery, University Hospitals Coventry and Warwickshire NHS Trust, Coventry, United Kingdom; 1 Department of Surgery, University Hospitals Coventry and Warwickshire NHS Trust, Coventry, United Kingdom; 2 >Department of Acute Medicine, Ajman University, Ajman, United Arab Emirates; 2 Department of Acute Medicine, Ajman University, Ajman, United Arab Emirates; 3 Department of Medicine, Chalmeda Ananda Rao institute of medical sciences, Telangana, India; 3 Department of Medicine, Chalmeda Ananda Rao institute of medical sciences, Telangana, India; 4 >Department of Cardiology, Manipal Hospitals West Bengal, India; 4 Department of Cardiology, Manipal Hospitals, West Bengal, India; 5 >Department of Acute Medicine, Royal Cornwall Hospital, Cornwall, United Kingdom; 5 Department of Acute Medicine, Royal Cornwall Hospital, Cornwall, United Kingdom; 6 Department of Pharmacology and Toxicology, College of Pharmacy, University of Hail, Hail, Saudi Arabia; 6 Department of Pharmacology and Toxicology, College of Pharmacy, University of Hail, Hail, Saudi Arabia; 7 Department of Medicine, White River Medical Center - White River Health, Arkansas, United States; 7 Department of Medicine, White River Medical Center - White River Health, Arkansas, United States; 8 Department of Medicine, Saveetha Dental College and Hospitals, Saveetha Institute of Medical and Technical Sciences (SIMATS), Saveetha University, Tamil Nadu, India; 8 Department of Medicine, Saveetha Dental College and Hospitals, Saveetha Institute of Medical and Technical Sciences (SIMATS), Saveetha University, Tamil Nadu, India; 9 >Department of Medicine, FMH Medical College, Punjab, Pakistan; 9 Department of Medicine, FMH Medical College, Punjab, Pakistan; 10 Department of Biochemistry, Quetta Institute of Medical Sciences (QIMS), Balochistan, Pakistan; 10 Department of Biochemistry, Quetta Institute of Medical Sciences (QIMS), Balochistan, Pakistan; 11 Department of Medicine, Scarborough General Hospital, Scarborough, United Kingdom; 11 Department of Medicine, Scarborough General Hospital, Scarborough, United Kingdom

**Keywords:** pediatric leukaemia, medication adherence, survivorship, fear of cancer recurrence

## Abstract

**Objectives:**

Childhood leukaemia patients who survived their treatment often experience Fear of Cancer Recurrence (FCR), which can affect Medication Adherence (MA), particularly in low-resource environments. This paper explored the relationship between FCR and adherence among pediatric survivors.

**Materials and Methods:**

A cross-sectional study was conducted in tertiary care pediatric oncology clinics in low-resource settings, involving 444 children diagnosed with Acute Lymphoblastic Leukaemia (ALL) or Acute Myeloid Leukaemia (AML). The FCR was assessed using the validated Fear of Cancer Recurrence Inventory-Short Form (FCRI-SF), and adherence was assessed using the validated Medication Adherence Rating Scale (MARS-10). The statistical tests were correlation test, independent t-tests, one-way ANOVA, and multiple linear regression.

**Results:**

The average FCRI-SF score was 27.0 +/- 2.6, and the average MARS-10 score was 15.1 +/-1.1. Medication adherence was significantly negatively associated with fear of cancer recurrence (r = -0.210, p < 0.001). Women had higher FCRI-SF (27.4 ± 2.9) than men (26.5 ± 2.4), and lower MARS-10 scores (14.8 ± 1.1 vs. 15.4 ± 1.0). The highest FCR (27.8 ± 2.4) and the lowest adherence (14.7 ± 1.0) were found in adolescents (15-17 years). Regression analysis revealed that FCR (B = -0.15, p = 0.001), female gender (B = -0.40, p = 0.001), treatment phase (B = -0.09, p = 0.003), and missed chemotherapy cycles (B = -0.07, p = 0.001) were significant predictors of poor adherence.

**Conclusion:**

FCR is a significant psychological risk factor of medication adherence in survivors of paediatric leukaemia. Psychosocial support and follow-up strengthening can be used to decrease non-adherence and treatment abandonment.

## Introduction

The most common cancer type in children is childhood leukaemia, and its onset is due to the abnormal growth of white blood cells in the bone marrow, which interferes with normal haematopoiesis^1)^. Childhood cancers such as leukaemia remain highly morbid and lethal and have few new treatments because of their rarity and biological diversity. In 2021, the reported childhood leukaemia cases were estimated worldwide to be 58,785, and the most significant number of cases and deaths were reported in Low Socio-Demographic Index (SDI) regions, with the highest number of cases affecting infants and boys^2, 3)^.

Acute leukaemias of childhood, particularly precursor B-cell Acute Lymphoblastic Leukaemia (ALL), tend to have an aetiological basis during the uterine period, with subsequent influences during the postnatal period, including infections and immune-associated genetic variations, which influence the disease^4)^. L-asparaginase is a fundamental treatment for pediatric ALL. Yet, immunogenicity, allergic reactions, and drug resistance limit its use, fueling the emergence of new variants and the development of individualised treatment approaches^5)^.

Survivorship also introduces a new dimension of concern, such as fear of cancer recurrence (FCR), that afflicts a significant percentage of childhood cancer survivors; about 43% of childhood cancer survivors report at least once, and 21% report persistently. Higher depressive symptoms and younger age of diagnosis correlate with higher FCR, which in most cases accompany long-term symptoms like pain and fatigue^6, 7)^.

Treatment abandonment, which entails the inability to finish therapy to control the disease, is a leading factor in treatment failure among children with cancer in low and middle-income countries^8)^. Medication non-adherence exists on a continuum, with treatment abandonment representing its most severe form. Minor lapses, such as missed appointments or incomplete follow-up, may progressively lead to treatment abandonment, particularly in low- resource pediatric oncology settings^9, 10)^. About 25% of children who have ALL withdraw therapy, significant ones being the costs of treatment, the belief in the incurability of the condition, problems in transportation, and the fear of adverse effects of treatment^11, 12)^.

In low-resource settings, limited access to follow- up care, psychosocial services, and health education may amplify fear of recurrence. These systemic gaps can exacerbate anxiety-driven non-adherence, increasing the risk of treatment interruption or abandonment^9)^. The interaction between fear of recurrence and treatment abandonment holds essential information to make effective survivorship care strategies. These factors can significantly impair the medical prognosis and mental health in resource-limited settings where psychosocial support is frequently low. Thus, the current research seeks to investigate the relationship between fear of recurrence and treatment abandonment in low- resource settings among pediatric patients who have survived leukaemia to establish the crucial psychosocial and systemic predictors that can be used in future interventions and policy formulation.

### Objectives

The main aim of the research is to investigate the rates and severity of FCR among survivors of childhood leukaemia in low-resource countries and to examine their correlation with treatment adherence. The study also seeks to determine the most important demographic, clinical, and psychosocial variables that may affect fear of recurrence and compliance with follow-up care. With knowledge of these determinants, the study aims to inform how survivors and their families face difficulties and where interventions, support programs, and policy modifications can be applied to curb treatment abandonment and improve overall survivorship in resource-constrained settings.

## Materials and Methods

### Ethical considerations

The institutional ethics review board of Scarborough General Hospital, Scarborough, United Kingdom (Approval No.: SGH-REC-2024-017) approved the study. Informed consent was obtained in written form from parents or legal guardians, and children aged seven or older provided assent. The involvement was voluntary, and confidentiality was ensured. Caregivers and children were assured that refusal or disengagement from the survey would not affect their ongoing medical care.

### Study approach and contextual setting

The cross-sectional study was conducted in tertiary care hospitals and pediatric oncology clinics in low-resource urban and semi-urban regions. The study sought to examine the relationship that exists between FCR and treatment abandonment among survivors of pediatric leukaemia.

### Study population and sampling

The patient population consisted of children aged between 5 and 18 years who had attained remission after being diagnosed with acute lymphoblastic or acute myeloid leukaemia. Only survivors who would not have a profound cognitive deficit or comorbidity that would have jeopardised participation were taken into consideration. A total of 444 participants, together with their primary caregivers, were recruited via convenience sampling to obtain a representative sample of survivors receiving follow-up care in low-resource settings. Convenience sampling was employed due to the absence of centralised survivorship registries, logistical constraints, and the need to maximise participant inclusion in low-resource pediatric oncology settings.

### Data collection instruments

Data were collected using three structured tools:

### Demographic questionnaire

A structured questionnaire was created to document age, gender, leukaemia type, treatment history, length of remission, family socioeconomic status, and distance.

### Medication adherence rating scale (MARS)

The MARS was developed by Thompson et al. (2000) to measure treatment adherence and consists of 10 items that assess medication adherence behaviours and attitudes. The items are rated using a dichotomy (Yes = 1, No = 0), and the overall score indicates better adherence. In children under 7 years of age, the primary caregiver would complete the MARS on behalf of the child; those 7 years or older would complete it themselves^13)^.

### Fear of cancer recurrence inventory-short form (FCRI-SF)

The FCRI-SF, developed by Simard and Savard in 2009, was used to measure FCR. This 9-item scale measures the severity of worry about cancer experienced in the previous month using a 5-point Likert scale (0 = never, 4 = almost always). The total score ranges from 0 to 36, with higher scores indicating greater fear of recurrence. In younger children, the caregiver provided the information on behalf of the child, but older children provided it themselves^14)^.

### Procedure

Data were collected through face-to-face interviews and from medical records. Parents or guardians provided informed consent, and children aged seven or older provided assent. For younger survivors, caregiver-reported responses were collected, as caregivers are primarily responsible for treatment supervision. Caregiver and self-reports were analysed together to reflect real-world adherence behaviours. Psychological support was provided to participants if they felt distressed when discussing recurrence or adherence.

### Scoring and interpretation

**MARS**: Total scores were summed; higher scores indicate greater adherence.

**FCRI-SF**: Scores were added and classified as low, moderate and high fear of recurrence according to recommended cut-offs.

The descriptive and subgroup analyses were conducted based on demographic variables.

### Data analysis

Data analysis was conducted in SPSS version 26. Descriptive statistics summarized demographic characteristics, fear of recurrence, and adherence scores. Pearson correlation was used to examine relationships between continuous variables (FCRI- SF and MARS-10). Independent samples t-tests and one-way ANOVA were used to compare groups, while multiple linear regression identified predictors of medication adherence. The level of statistical significance was set at p < 0.05.

## Results

### Summary of participant demographic characteristics

Table 1 summarises the demographics of 444 children with leukaemia. The majority of the respondents were teenagers aged 15-17 years (297, 66.9%) and male (257, 57.9%). The children mostly attended higher secondary school or college (115, 25.9% or high school (Grades 910; 106, 23.9%), and their living conditions were in urban (195, 43.9%) or rural areas (148, 33.3%). The number of acute myeloid leukaemia (AML, 268, 60.4%), acute lymphoblastic leukaemia (ALL, 176, 39.6%), and 153 (34.5%) were diagnosed within six months. The distribution of treatment stages was quite balanced, but the response to chemotherapy was not homogeneous, and the vast majority of children reported mild to severe side effects.

**Table 1:** Demographic characteristics of participants (N = 444)

Variable	f (N)	%		Variable	f (N)	%
Child's age				25-34 years	150	33.8
5-7 years	8	1.8		35-44 years	100	22.5
8-11 years	41	9.2		45-54 years	75	16.9
12-14 years	98	22.1		55 years or above	59	13.3
15-17 years	297	66.9		Parent/Caregiver educational level		
Child’s gender				No formal education	108	24.3
Male	257	57.9		Primary (Grades 1-5)	128	28.8
Female	187	42.1		Middle (Grades 6-8)	73	16.4
Child’s educational level				High school (Grades 9-10)	49	11.0
Not attending school	63	14.2		Higher secondary/college	54	12.2
Primary (Grades 1-5)	90	20.3		University/professional	32	7.2
Middle (Grades 6-8)	70	15.8		Leukaemia type		
High school (Grades 9-10)	106	23.9		ALL (Acute lymphoblastic leukaemia)	176	39.6
Higher secondary/college	115	25.9		AML (Acute myeloid leukaemia)	268	60.4
Residence place				Time since diagnosis		
Urban	195	43.9		< 6 months	153	34.5
Semi-urban	101	22.7		6-12 months	94	21.2
Rural	148	33.3		1-2 years	79	17.8
Distance to treatment center				> 2 years	118	26.6
< 5 km	99	22.3		Current treatment phase		
5-10 km	128	28.8		Induction	121	27.3
11-25 km	99	22.3		Consolidation/Intensification	120	27.0
> 25 km	118	26.6		Maintenance	96	21.6
Travel time to hospital				Completed	107	24.1
< 30 minutes	137	30.9		Number of chemotherapy cycles missed		
30-60 minutes	141	31.8		None	191	43.0
1-2 hours	85	19.1		1 therapy	81	18.2
>2 hours	81	18.2		2 therapies	64	14.4
Parent/Caregiver relationship to child				> 2 therapies	108	24.3
Mother	200	45.0		Experience of treatment side effects		
Father	180	40.5		None	32	7.2
Other relative/guardian	64	14.4		Mild	158	35.6
Parent/Caregiver age				Moderate	110	24.8
< 25 years	60	13.5		Severe	144	32.4

Note. f = frequency, % = percentage; Values are presented as N (%), N = 444; No statistical comparisons were performed for demographic variables in this table.

### Correlation between fear of cancer recurrence and medication adherence

Table 2 shows a significant negative correlation between fear of cancer recurrence (FCRI-SF) and medication adherence (MARS-10) (r = -0.210, t = -4.52, df = 442, p < 0.001). This indicates that higher fear of recurrence was associated with lower medication adherence. In this study, non-adherence is used as a proxy for treatment abandonment in pediatric leukaemia survivors.

**Table 2:** Association between fear of cancer recurrence (FCRI-SF) and medication adherence (MARS-10) (N = 444)

Variables	r	t(df)	p
Total fear of cancer recurrence inventory-short form (FCRI-SF)	-	-	-
Total medication adherence rating scale (MARS)	-0.210	-4.52 (442)	<0.001**

Note. Values represent Pearson correlation coefficients (r) between continuous variables; N = 444; p < 0.001 (2-tailed) was considered statistically significant and is denoted with double asterisks (**).

### Gender-based differences in fear of cancer recurrence and medication adherence

Table 3 reveals that there was a significant gender difference in both fear of recurrence of cancer and medication adherence among the 444 respondents. Females reported slightly higher fear of cancer recurrence (M = 27.4 ± 2.9) than males (M = 26.5 ± 2.4), which was statistically significant (t = -3.10, p = 0.002, Cohen’s d = 0.38). Conversely, males showed higher medication adherence (M = 15.4 ± 1.0) than females (M = 14.8 ± 1.1; t = 4.20, p < 0.001, Cohen’s d = 0.50). These results indicate a small-to-moderate gender difference in both FCR and adherence.

**Table 3:** Gender differences in fear of cancer recurrence (FCRI-SF) and medication adherence (MARS) (N = 444)

Variables	Male (N=257; 57.9%); (M ± SD)	Female (N=187; 42.1%); (M ± SD)	*t*	*p*	95% CI		Cohen's *d*
					LL	UL	
Total fear of cancer recurrence inventory-short form (FCRI-SF)	26.50 ± 2.40	27.40 ± 2.90	-3.10	0.002**	-1.46	-0.34	0.38
Total medication adherence rating scale (MARS)	15.40 ± 1.00	14.80 ± 1.10	4.20	< 0.001**	0.31	0.82	0.50

Note. Values are presented as Mean ± Standard Deviation; Independent samples t-tests were conducted to compare participants of both male and female groups; Group sizes are shown as N (%); Reported statistics include p-values, t-values, 95% Confidence Intervals (CI), and effect sizes (Cohen’s d); A p-value < 0.001 was considered statistically significant, N = 444.

### Visual comparison of gender differences in FCRI-SF and MARS scores

Figure 1 shows gender variations in FCRI-SF and MARS scores among the 444 participants. Females reported slightly higher fear of cancer recurrence (FCRI-SF) than males, while males demonstrated higher medication adherence (MARS). These differences were statistically significant, indicating a small-to-moderate effect of gender on both FCR and adherence.

**Figure 1:**
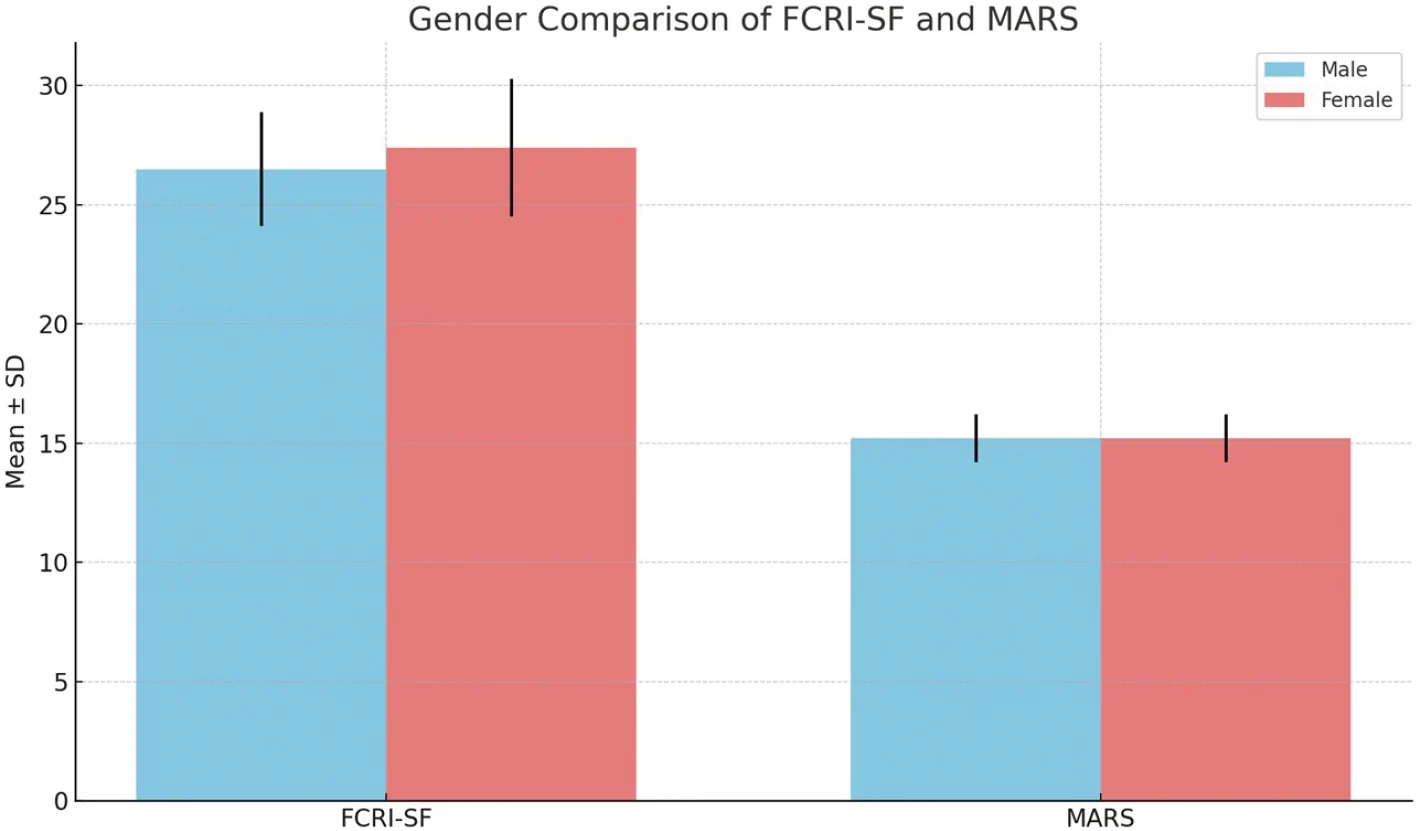
Gender differences in FCRI-SF and MARS mean scores (N = 444). Figure created by the authors

### Age-related differences in fear of cancer recurrence and medication adherence

Age-related differences in FCR and medication adherence are presented in Table 4. FCR increased with age (F(3, 440) = 11.02, p < 0.001, η^2^ = 0.070), with adolescents (15-17 years) reporting the highest fear. Medication adherence declined slightly with age (F(3, 440) = 3.90, p = 0.009, η^2^ = 0.026), suggesting a minimal age effect. These findings indicate that older children and adolescents experience greater fear of recurrence and slightly lower adherence.

**Table 4:** Age differences in fear of cancer recurrence (FCRI-SF) and medication adherence (MARS-10) among children and adolescents (N = 444)

Variables	5-7 years (N = 8; 1.8); (M ± SD)	8-11 years (N = 41; 9.2); (M ± SD)	12-14 years (N = 98; 22.1); (M ± SD)	15-17 years (N = 297; 266.9); (M ± SD)	F(3,440)	p	η^2^
Total fear of cancer recurrence inventory-short form (FCRI-SF)	25.00 ± 2.50	26.20 ± 2.60	27.10 ± 2.70	27.80 ± 2.40	11.02	<0.001***	0.070
Total medication adherence rating scale (MARS)	15.30 ± 0.90	15.10 ± 1.00	14.90 ± 1.05	14.70 ± 1.00	3.90	0.009**	0.026

Note. Data are presented as Mean ± Standard Deviation (M ± SD); Group sizes are shown as N (%); One-way ANOVA was conducted to examine the effect; All comparisons were significant at p < 0.01, < 0.001; η^2^ represents partial eta-squared effect size.

### Predictors of medication adherence based on psychological, demographic, and clinical factors

Table 5 presents the results of multiple regression-based prediction of medication adherence among children with leukaemia. Multiple linear regression (Table 5) showed that higher FCRI-SF scores significantly predicted lower medication adherence (B = -0.15, β = -0.35, t = -5.00, p < 0.001). Older age predicted greater adherence (B = 0.10, β = 0.20, t = 4.00, p < 0.001), while female gender predicted lower adherence (B = -0.40, β = -0.18, t = -3.33, p = 0.001). Clinical variables also influenced adherence: leukaemia type (B = 0.22, β = 0.12, p = 0.006), current treatment phase (B = -0.09, β = -0.12, p = 0.003), and number of missed chemotherapy cycles (B = -0.07, β = -0.11, p < 0.001) were significant predictors. Overall, psychological, demographic, and treatment-related factors contribute to medication adherence among pediatric leukaemia survivors.

**Table 5:** Multiple regression predicting medication adherence (MARS-10) from fear of cancer recurrence, age, gender, and clinical variables (N = 444)

Predictor	B	SE	β	t	p	95% CI lower	95% CI upper
(Constant)	13.50	0.50	-	27.00	< 0.001***	12.52	14.48
Total FCRI-SF	-0.15	0.03	-0.35	-5.00	< 0.001***	-0.21	-0.09
Child age (years)	0.10	0.03	0.20	4.00	< 0.001***	0.05	0.15
Child gender (female)	-0.40	0.12	-0.18	-3.33	0.001**	-0.64	-0.16
Leukemia type	0.22	0.08	0.12	2.75	0.006**	0.06	0.38
Current treatment phase	-0.09	0.03	-0.12	-3.00	0.003**	-0.15	-0.03
Number of chemotherapy cycles missed	-0.07	0.02	-0.11	-3.50	< 0.001***	-0.11	-0.03

Note. Multiple linear regression was conducted to identify predictors of Medication Adherence (MARS-10) Scores; Values include unstandardized coefficients (B), 95% confidence intervals (CI), standard error (SE), standardised beta coefficients (β), and p-values; **= p < 0.01, ***= p < 0.001 was considered statistically significant, N = 444.

## Discussion

In the current research, the FCR was a significant predictor of poor medication adherence among patients with leukaemia, and this meant that the anxiety related to the disease returning may decrease adherence to medications. This is in line with the earlier studies, which reported fear of recurrence to be strongly correlated with medication beliefs and illness perceptions, which indicate that the psychological aspects can affect the adherence behaviour among all cancer populations^15)^.

The observed gender differences suggest that female survivors may experience greater emotional vulnerability, which could partially explain lower adherence behaviors, and the effect was slight-to-moderate, consistent with previous findings of slight but consistent gender differences in FCR^16)^. Consequently, medication adherence was higher in male participants compared to female participants, which agrees with the literature that female cancer survivors tend to report cost-related or overall non- adherence^17)^.

Age was also correlated with FCR and adherence. The FCR was highest among adolescents and lowest among the youngest children (5-7 years), which aligns with a developmental approach, in which older children tend to be more aware of potential health risks^18)^. In line with this, compliance was minimally age-dependent, with adolescents less compliant than younger children, suggesting decreased parental supervision and increased autonomy^19)^.

Greater FCR was a predictor of worse medication adherence, with females showing slightly lower adherence, and age affecting adherence patterns, consistent with prior research^15, 17, 19)^. Clinical factors also influenced adherence patterns. The type of leukaemia was a significant predictor of adherence, and some subtypes showed slightly higher MARS- 10 scores, as previously reported^20)^. The level of medication adherence also decreased in the latter, more intensive stages of treatment, probably because of the increased weight of home-based maintenance treatment and issues with caregivers^21)^. Lastly, lower adherence was linked to absenteeism from chemotherapy sessions, highlighting the clinical significance of continuous oral maintenance therapy in pediatric ALL^22)^.

Together, these findings underscore the synergies among psychological, demographic, and clinical factors in medication compliance among children with leukaemia. These findings highlight the importance of integrating routine screening for fear of recurrence into survivorship care, particularly for adolescents and female survivors. Targeted psychosocial interventions may help mitigate anxiety-driven non-adherence.

### Limitations of our study and future recommendations

This research has several limitations that must be taken into account when analysing the outcomes. First, the cross-sectional design does not allow for causal inference regarding the relationship between fear of recurrence and adherence. Second, self- report scales, particularly when an adult completes them on behalf of young children, can be vulnerable to response and social desirability bias. Third, convenience sampling of hospitals limits the generalizability of the results to all low-resource areas. Lastly, some contextual variables, including financial burden, cultural attitudes toward cancer and access to transportation, were also not examined but could be significant contributors to adherence and psychological distress. Additionally, medication adherence was self-reported, which may introduce recall or social desirability bias. Finally, the study did not include a qualitative exploration of psychosocial factors, limiting the understanding of underlying barriers to adherence.

Future studies ought to use longitudinal designs to monitor fear of recurrence and adherence patterns throughout and after treatment. Psychosocial intervention, including counselling, family-based support programs and structured survivorship education, needs to be considered culturally adapted to decrease FCR and enhance adherence. Furthermore, health systems in low-resource countries should consider approaches such as engaging community health workers, providing telemedicine follow-ups, and implementing financial or logistical support interventions to reduce treatment abandonment. Additional information about the family's experience and barriers to regular care could also be obtained through qualitative research.

## Conclusion

Overall, it is concluded that FCR is associated with poor medication adherence among low-resource setting pediatric survivors of leukaemia. The research demonstrates the interplay among psychological, demographic, and clinical factors in adherence behaviour, as well as the importance of integrative survivorship care. The treatment of FCR in childhood leukaemia survivors by identifying it early, supporting it using psychosocial means, and improving the system can potentially decrease the rate of treatment abandonment and improve overall long-term survival. At a policy level, strengthening survivorship programs with integrated psychosocial support and adherence monitoring is essential to reduce treatment abandonment in low-resource pediatric oncology settings.

## Data availability

The datasets generated and analysed during the current study are available from the corresponding author on reasonable request.

## Author contributions

SS and AFM conceived and designed the study and supervised the overall project. DN and PC contributed to conceptual support, data interpretation, and critical revision of the manuscript. MV and SA were responsible for data analysis, manuscript preparation, and final approval. KMT and SHK contributed to the literature review and referencing. KWM assisted with data validation and statistical review. KS coordinated formatting, proofreading, and preparation of tables and figures. All authors read and approved the final manuscript.

## Conflicts of interest statement

The authors declare that there are no conflicts of interest.
